# Biocompatible Nanoparticles as a Platform for Enhancing Antitumor Efficacy of Cisplatin–Tetradrine Combination

**DOI:** 10.1186/s11671-021-03511-4

**Published:** 2021-04-14

**Authors:** Fangcen Liu, Xinyue Wang, Qin Liu, Huan Zhang, Li Xie, Qin Wang, Lin Li, Rutian Li

**Affiliations:** 1 Department of Pathology, Nanjing Drum Tower Hospital, The Affiliated Hospital of Nanjing University Medical School, Nanjing, China; 2 The Comprehensive Cancer Centre of Nanjing Drum Tower Hospital, The Affiliated Hospital of Nanjing University Medical School, Nanjing, China; 3 Center for Personalized Medicine, Linköping University, 58183 Linköping, Sweden

**Keywords:** Copolymer, Nanoparticles, Combination therapy, Cisplatin, Tetradrine

## Abstract

Combination therapy has been a standard strategy in the clinical tumor treatment. We have demonstrated that combination of Tetradrine (Tet) and Cisplatin (CDDP) presented a marked synergistic anticancer activity, but inevitable side effects limit their therapeutic concentration. Considering the different physicochemical and pharmacokinetic properties of the two drugs, we loaded them into a nanovehicle together by the improved double emulsion method. The nanoparticles (NPs) were prepared from the mixture of poly(ethyleneglycol)–polycaprolactone (PEG–PCL) and polycarprolactone (HO-PCL), so CDDP and Tet can be located into the NPs simultaneously, resulting in low interfering effect and high stability. Images from fluorescence microscope revealed the cellular uptake of both hydrophilic and hydrophobic agents delivered by the NPs. In vitro studies on different tumor cell lines and tumor tissue revealed increased tumor inhibition and apoptosis rates. As to the in vivo studies, superior antitumor efficacy and reduced side effects were observed in the NPs group. Furthermore, ^18^FDG-PET/CT imaging demonstrated that NPs reduced metabolic activities of tumors more prominently. Our results suggest that PEG–PCL block copolymeric NPs could be a promising carrier for combined chemotherapy with solid efficacy and minor side effects.

**Supplementary Information:**

The online version contains supplementary material available at 10.1186/s11671-021-03511-4.

## Introduction

With the development of tumor comprehensive therapy, platinum compounds play an important role in the treatment of various cancers, among which cisplatin (CDDP) is widely used in the clinic [1, 2]. Nowadays, CDDP is still significant when combined with tumor immunotherapy, showing a favorable effect on malignant tumors such as non-small cell lung cancer (NSCLC) [3]. However, these chemotherapeutic agents always achieve antitumor activity at the expense of undesirable toxicity [4], so therapeutic agents that can reduce toxicity and enhance the efficacy of chemotherapy have been ceaselessly explored [5, 6]. Tetrandrine (Tet) is a member of bis-benzylisoquinoline alkaloid [7], showing satisfactory effect in sensitization to chemotherapy. Our previous work has demonstrated that combination of Tet and CDDP does have a marked synergistic anticancer activity by inhibiting the expression of chemotherapeutic agent-associated genes, including the excision repair cross-complementation group 1 (ERCC1), Thymidylate Synthase (TS), β-tubulin III, etc [8]. However, the clinical application of Tet suffers from its poor water-solubility and low oral bioavailability [7]. Moreover, hydrophilic CDDP distributes most readily in the extracellular matrix (ECM), while hydrophobic Tet penetrate lipid membranes can be transported through cells, so the two drugs can't really work together. Therefore, it is essential to find a way that can effectively deliver the two drugs simultaneously, improving the antineoplastic effect with reduced side effects.

Recent advancements in the field of nanotechnology manage novel approaches for diagnosis and treatment of cancer [9, 10]. Nanoparticles (NPs) constructed with amphiphilic copolymers, especially polyethylene glycol (PEG), are known for their abilities to reduce adherence of serum protein and prevent uptake by the reticuloendothelial system (RES) [11]. If used to carry hydrophobic drugs, the nanocarriers increase solubility tactfully and prolong as well as increase drug residency in blood circulation and tumor [12]. With the clinical use of copolymeric NPs, the biocompatibility of the NPs attracts more and more attention. For example, recent findings revealed that some injected titanium dioxide, silica, and gold nanoparticles accelerate intravasation and extravasation of cancer cells in animal models [13]. The NPs prepared from nanomaterials with good biocompatibility and safety, especially those approved by FDA, are better candidates for drug carriers.

Preliminarily, we have managed to construct CDDP-loaded NPs [14, 15] and Tet-loaded NPs using polyethylene glycol–poly(caprolactone) (PCL–PEG) [16], whose in vivo antitumor effects have both been demonstrated. In this study, we used PEG-PCL for the co-delivery of CDDP and Tet. With a near-neutral charge, PEG forms the hydrophilic shell of a nanoparticle, which hides antigenic epitopes and prevents immunologic reaction [14]. Images from fluorescence microscope demonstrated the cellular can uptake both hydrophilic and hydrophobic agents delivered by the NPs. The results of in vitro studies, not only on different tumor cell lines, but also on tumor tissues, revealed CDDP-Tet NPs inhibit tumor growth more effective than the free drugs. When researched in vivo, increased antitumor efficacy and decreased side effects were observed in the NPs group. Moreover, ^18^FDG-PET/CT imaging showed the poorest metabolism rate of the tumor in the NPs group, and thereby indicating the capability of CDDP-Tet NPs to retard tumor growth.

## Methods

### Materials

#### Reagents and Cells

CDDP (molecular formula PtCl_2_(NH_3_)_2_) was purchased from Shandong Boyuan Pharmaceutical Co. Ltd. (Jinan China) [15]. Tetrandrine (molecular formula C_38_H_42_N_2_O_6_) was obtained as a powder with a purity of > 98% from Jiangxi Yibo Pharmaceutical Development Company (Jiangxi, China) [16]. Methoxypolyethyleneglycol [MePEG; weight-average molecular weight (Mw = 4 kDa; Nanjing Well Chemical Co. Ltd. China] was dehydrated by azeotropic distillation with toluene and then vacuum dried at 50 °C for 12 h before use. ε-Caprolactone (ε-CL; Aldrich, USA) was purified by drying over CaH_2_ at room temperature followed by distillation under reduced pressure. Polyvinyl alcohol (PVA; polymerization degree = 500, alcoholization degree = 88%; Shanghai Dongcang International Trading Co. Ltd., China) and stannous octoate (molecular formula SnCl2) (Sigma) were used as received. RPMI 1640 medium (Gibco, USA), calf blood serum (Lanzhou Minhai Bioengineering, China), and dimethylthiazoly-2,5-diphenyltetrazolium bromide (MTT; Amersco, USA) were used as received.

Human well-differentiated gastric cancer cell line MKN_28_, human colorectal adenocarcinoma cell line LoVo, human cervical cancer cell line Hela, and murine hepatoma cell line H_22_ were obtained from Shanghai Institute of Cell Biology (Shanghai, China). All cell lines were propagated in RPMI 1640 medium, supplemented with 10% bovine serum, penicillin (100 U/mL)-streptomycin (100 g/mL), pyruvate, glutamine, and insulin at 37 °C in a water-saturated atmosphere with 5% CO_2_.

#### Synthesis of the Copolymers

As we previously described [16, 17], mPEG–PCL and HO-PCL copolymers were synthesized by a ring opening copolymerization. Briefly, predetermined amount of ε-CL was added into a polymerization tube containing PEG and a small amount of stannous octoate (0.1% wt/wt). The tube was then connected to a vacuum system, sealed off, and placed in an oil bath at 130 °C for 48 h. For the synthesis of HO-PCL copolymers, predetermined amount of ε-CL and stannous octoate were added into a polymerization tube without dry. After the polymerization was complete, the crude copolymers were dissolved with chloroform and precipitated into an excess amount of cold methanol to remove the unreacted monomer and oligomer. The precipitates were then filtered and washed with water several times before thoroughly dried at reduced pressure.

#### Preparation of CDDP-Tet-Loaded Nanoparticles

**Fig. 1:**
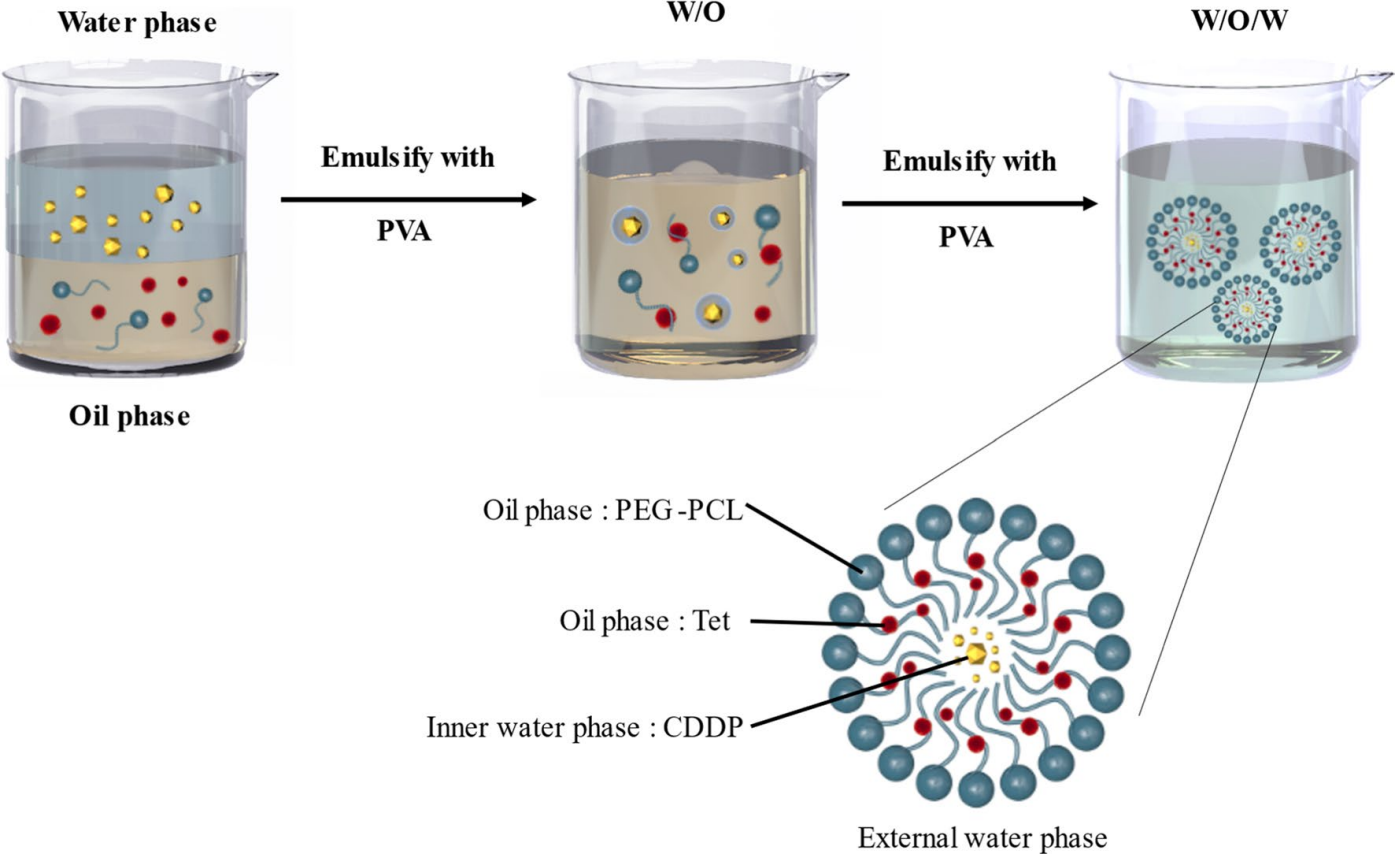
The scheme of CDDP-Tet NPs preparation

Predetermined amount of CDDP and Tet-loaded nanoparticles were prepared by double emulsion (DE) method [14, 18]. CDDP and Tet was emulsified with 1 mL of dichloromethane (DCM) solution containing 5 mg of mPEG–PCL and 15 mg of HO-PCL, by sonication for 15 s (15 W) in an ice bath (solution W1). Then, 4 mL of 3% (w/v) PVA solution W2 was added and sonicated for 30 s to make a W1/O/W2 emulsion. The double emulsion was diluted into 50 mL of 0.3% (w/v) PVA aqueous solution and DCM was evaporated under vacuum. The obtained nanoparticles were collected, washed, and freeze dried (Fig. 1).

#### Drug-Loading Content and Encapsulation Efficiency

To determine the drug loading content, the freeze-dried CDDP–Tet-loaded NP powder was dissolved in dimethylformamide (DMF), and 30 L of this solution was mixed with 30 L of 2 mmol/L HCL followed by the addition of 2.94 mL of 0.2 mmol/L SnCl_2_ solution in 2 mmol/L HCL. The absorbance at 403 nm was measured after 1 h with reference to a calibration curve using a Shimadzu UV-1205 Spectrophotometer (Kyoto, Japan). The total amount of the drug in the NPs could then be calculated. The drug loading content and encapsulation efficiency were, respectively, obtained by Eqs. (1) and (2):

1 $${\text{Drug loading content}}\, (\% ) = \frac{{{\text{Weight of the drug in nanoparticles}}}}{{{\text{Weight of the nanoparticles}}}} \times 100\,(\% )$$

2 $${\text{Encapsulation efficiency }}\,(\% ) = \frac{{{\text{Weight of the drug in nanoparticles}}}}{{{\text{Weight of the feeding drug}}}} \times 100\,(\% )$$

### In Vitro Cytotoxicity of the Nanoparticles and the Biocompatibility Study

#### Cellular Uptake Studies

On the basis of our previous work [15], LoVo cells were placed on the cover of 6-pore plate with about 5 × 10^5^ cells each pore and were incubated for 24 h (37 °C, 5% CO_2_). NPs with rhodamine B (21 μg/mL) were added in the pores. After incubation for 2 h, the pate was washed with 4 °C and 37 °C of PBS for 3–4 times each. LoVo cells on cover slips were then subjected to fluorescence microscope.

### Cytotoxicity Assay

The in vitro cytotoxicity of the drugs was determined by standard MTT assays using MKN28 and H_22_ cell lines. Briefly, cells were seeded in a 96-well plate at a density of 5000 cells per well 24 h prior to the assay. Then cells were exposed to a series of doses of free CDDP, free Tet, free CDDP plus Tet and CDDP-Tet-loaded nanoparticles. After incubation, 20 μL of 5 mg/mL MTT solution was added to each well and the plate was incubated for 4 h, allowing the viable cells to transform the yellow MTT into dark-blue formazan crystals, which were dissolved in 200 μL of dimethyl sulphoxide (DMSO). The optical density (OD) of each well was measured by an ELISA reader (ELX800 Biotek, USA) using test and reference wavelengths of 490 and 630 nm, respectively. Cell viability was determined by the following formula:

3 $${\text{Cell viability}}\,(\% ) = \frac{{{\text{OD (test well)}}}}{{{\text{OD (reference well)}}}} \times 100\,(\% )$$

The in vitro compatibility of the blank nanoparticles was also determined by MTT assays using MKN_28_ and H_22_ cell lines. All the results obtained from MTT assays were confirmed by repeating the experiment on at least three independent occasions and testing in triplicate each time.

### Apoptosis Assay

Annexin V-FITC Kit (Bender MedSystem, USA) was adopted to examine the alteration of cell apoptosis rates. MKN_28_ cells were subjected to a 6 cm culture dish for 24 h. Culture media were replaced with 1 μg/mL free CDDP, 1 μg/mL CDDP-loaded NPs, and 200 μg/mL blank NPs, respectively. The control group was replaced with culture media without drugs. Enzyme was added following 48 h of culture. The cells were then collected, washed 2 times with PBS, and resuspended in 100 μL buffer. Annexin V 5 μL and PI 1 μL were added in turn, mixed, and stood for 15 min at room temperature without exposure to light. 400 mL buffer was added and was undergone FCM (BD FACS CantoTM, USA) process for cell apoptosis rates analysis.

### Histoculture Drug Response Assay (HDRA)

The HDRA was performed according to our previous studies [14, 19]. Briefly, male ICR mice were injected subcutaneously at both axillary spaces with 4–6 × 10^6^ H_22_ cells in saline. When the tumors reached 400–600 mm^3^ in volume, the mice were sacrificed by cervical dislocation, and fresh specimens were sampled, washed twice in saline, immersed in Hank’s solution, and divided into pieces with weights of approximately 15 mg. The tissue samples were placed in a 24-well plate into which square gelatin sponges with dimensions of 1 cm had been immersed in RPMI 1640 medium supplemented with 20% fetal calf serum and amikacin sulfate (100 IU/mL) containing free CDDP or CDDP-loaded NPs at two different concentrations. Four tumor specimens were incubated without any drug as a control. The tissues were then cultured for 7 days at 37 °C 5% CO_2_. A mixed solution of Type I collagenase (100 L, 0.6 mg/mL) and MTT (100 L, 5 mg/mL) in 100 mg/mL sodium succinate was added. After incubation for another 24 h, MTT formazan was extracted by 1 mL DMSO, and 100 L of solution from each well was transferred to the wells of a 96-well microplate. The OD of each well in the microplate was measured using the ELISA reader with test and reference wavelengths of 490 and 630 nm, respectively. The viabilities of the tissues were calculated according to the following formula (4):

4 $${\text{Tissue viability}}\,(\% ) = \frac{{{\text{OD (test)}}/{\text{Weight (test)/mg}}}}{{{\text{OD (control)}}/{\text{Weight (control)/mg}}}} \times 100\,(\% )$$

### In Vivo Antitumor Efficacy

#### Tumor Volume Measurement

Male ICR mice with weight between 18 and 20 g were implanted with murine hepatoma cell line H_22_ and used to qualify the relative efficacy of CDDP-Tet-loaded NPs. Raised under specific pathogen-free (SPF) circumstances, the mice were performed in compliance with guidelines approved by the Animal Care Committee at Drum Tower Hospital. 0.2 mL of cell suspension containing 4–6 × 10^6^ H_22_ cells were injected subcutaneously into the left axillary space of the mice. The mice were divided into 6 groups: control group (saline), blank NPs group, free CDDP (3 mg/kg) group, free CDDP plus Tet (CDDP 3 mg/kg + Tet 7.2 mg/kg) group, and CDDP-Tet-loaded NPs (CDDP 3 mg/kg + Tet 7.2 mg/kg) group. Each group was comprised of 6 mice. Treatments were started after 7–8 days of implantation, and the day was designated as ‘‘Day 0.” Each animal was weighed at the time of treatment so that dosages could be adjusted to achieve the reported mg/kg doses. Animals were also weighed every other day throughout the experiment.

### Immunofluorescence Assay

The tumor tissues from the mice in control group and that received free CDDP plus Tet and CDDP-Tet NPs were selected for histology observation on the 21st day after treatment. The tumors were dissected and fixed in 10% neutral buffered formalin, routinely processed into paraffin, sectioned at a thickness of 5 mm. The tissue sections were observed under a Zeiss LSM510 Meta confocal microscope after the samples were stained with (40,6-diamidino-2-phenylindole) (DAPI, blue) and terminal deoxynucleotidyl transferase dUTP nick end labeling (TUNEL, green) [20].

### ^18^FDG-PET/CT Imaging

Mice of free CDDP plus Tet group and CDDP-Tet-loaded NPs group were then received PET/CT imaging on day 6 after treatment. 4 h of fasting were conducted before tracer injection. 14.8 MBq (400 lCi) of ^18^F-FDG was injected via the tail vein as a radiotracer. The images were produced with a combined PET/CT scanner (Jemini JXL, Philips, USA). High-resolution PET images and the same field of CT view were acquired with the mice 45 min after the administration of ^18^F-FDG. PET images were corrected for attenuation and scatter on the basis of the CT data. The image fusion was performed by an automatic image fusion system, using vendor-supplied software. Maximum FDG uptake values in the tumor were obtained for the standard uptake value (SUV) calculations, applying corrections for body weight and injected activity.

### Statistical Methods

Statistical analyses of data were done using Student’s *t* test. The data are listed as mean ± SD, and values of *P* < 0.05 were accepted as a statistically significant difference.

## Results

### Copolymer Synthesis and Characterization

According to our prior study, we synthesized the NPs with PEG–PCL and HO-PCL(the optimal ratio was 1:3) [14]. The diameter, polydispersity, number-average molecular weight (Mn), and weight-average molecular weight (Mw) of the optimal PEG–PCL copolymeric NPs are shown in Additional file 1: Table S1. When loaded with CDDP and Tet, drug loading content and encapsulation efficiency were also measured (Additional file 1: Table S2), which are similar to those of CDDP-loaded PEG–PCL copolymeric NPs. However, the drug loading content and loading efficiency of Tet are less than those of Tet-loaded PEG–PCL copolymeric NPs, which may have something to do with HO-PCL components.

Through dynamic light scattering (DLS), the diameter of CDDP-Tet-loaded PEG–PCL copolymeric NPs was 359.1 ± 5.3 nm with a polydispersity around 0.231. When observed by TEM and AFM (Additional file 1: Fig S1a and S1b), the NPs presented regular spherical shape and similar sizes which in coincidence with the data from DLS. Also, small droplets are dispersed in the nanoparticles, which confirms nanoparticles are indeed the structure of double emulsion.

### Cellular Uptake of CDDP-Tet-Loaded NPs

**Fig. 2:**
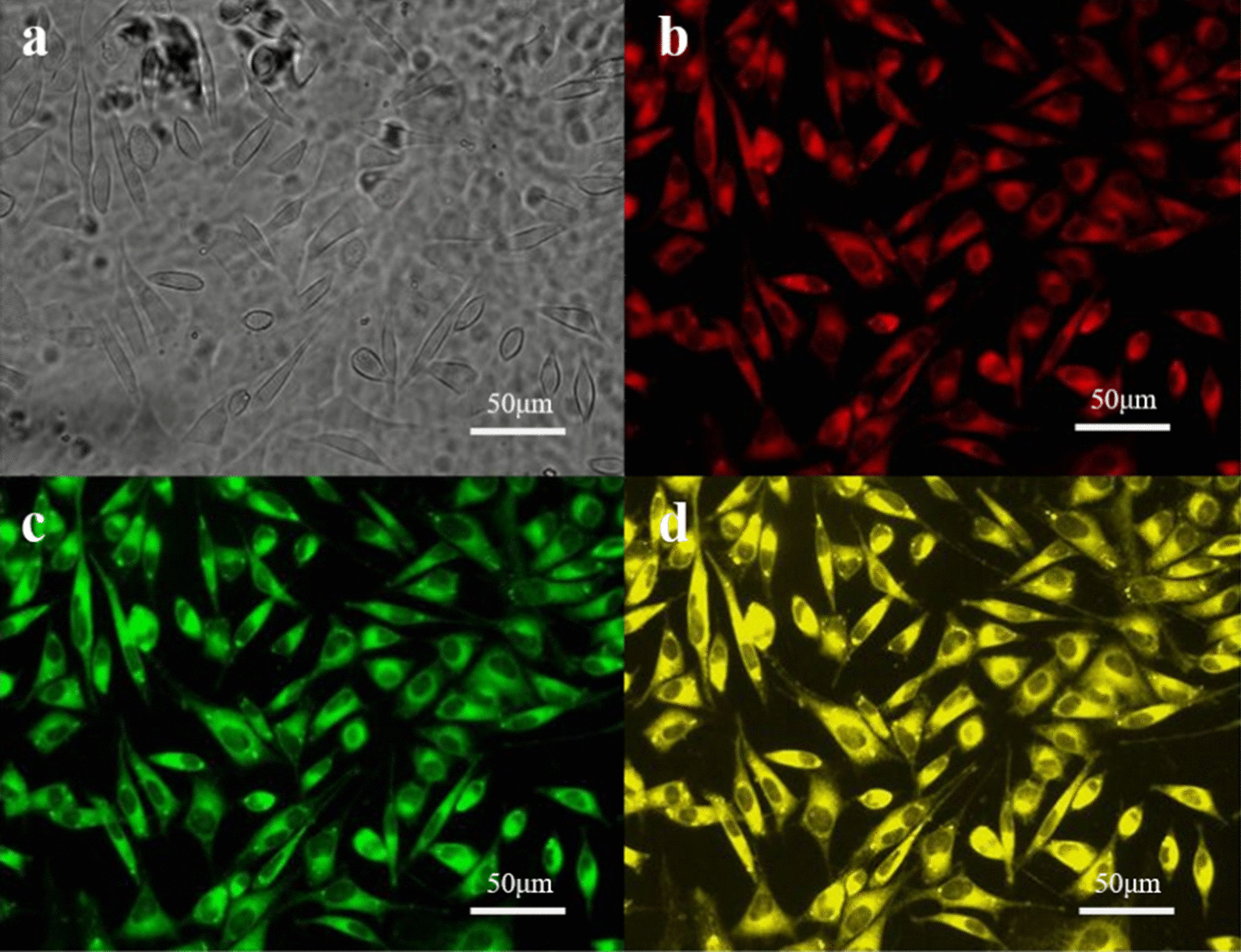
Photographs of LoVo cells after 2 h of staining with NPs loaded with rhodamine B and coumarin-6 (200 × ; bar: 50 um). **a** Cell morphology under the light microscopy, **b** cell morphology under fluorescence microscope(PI fluorescence channel), **c** cell morphology under fluorescence microscope(FITC fluorescence channel), and **d** cell morphology under fluorescence microscope(PI/FITC dual fluorescence channel)

Particles loading with fluorescent dyes have been applied as a common method to explore cellular uptake, which realize visual and real-time detection. We have demonstrated that the dye-loaded NPs can enter the cells through endocytosis. In this study, rhodamine-B is hydrophilic and can be detected in PI fluorescence channel, which are used to simulate CDDP. As the simulation of Tet, coumarin-6 is hydrophobic, whose signal can be received in the FITC fluorescence channel. After being co-incubated with NPs loaded with coumarin-6 and rhodamine-B for 2 h, LoVo cells were detected through fluorescence microscopy and optical lens (200 ×). As can be seen in Fig. 2, fluorescence signals can be detected in PI fluorescence channel, the FITC fluorescence channel, as well as the PI/FITC dual fluorescence channel. The results confirmed that the NPs can carry both coumarin-6 and rhodamine-B to tumor cells at the same time, based on which we can infer that CDDP and Tet can be loaded in the NPs and absorbed by tumor cells simultaneously.

### In Vitro Cytotoxicity of the Nanoparticles

**Fig. 3:**
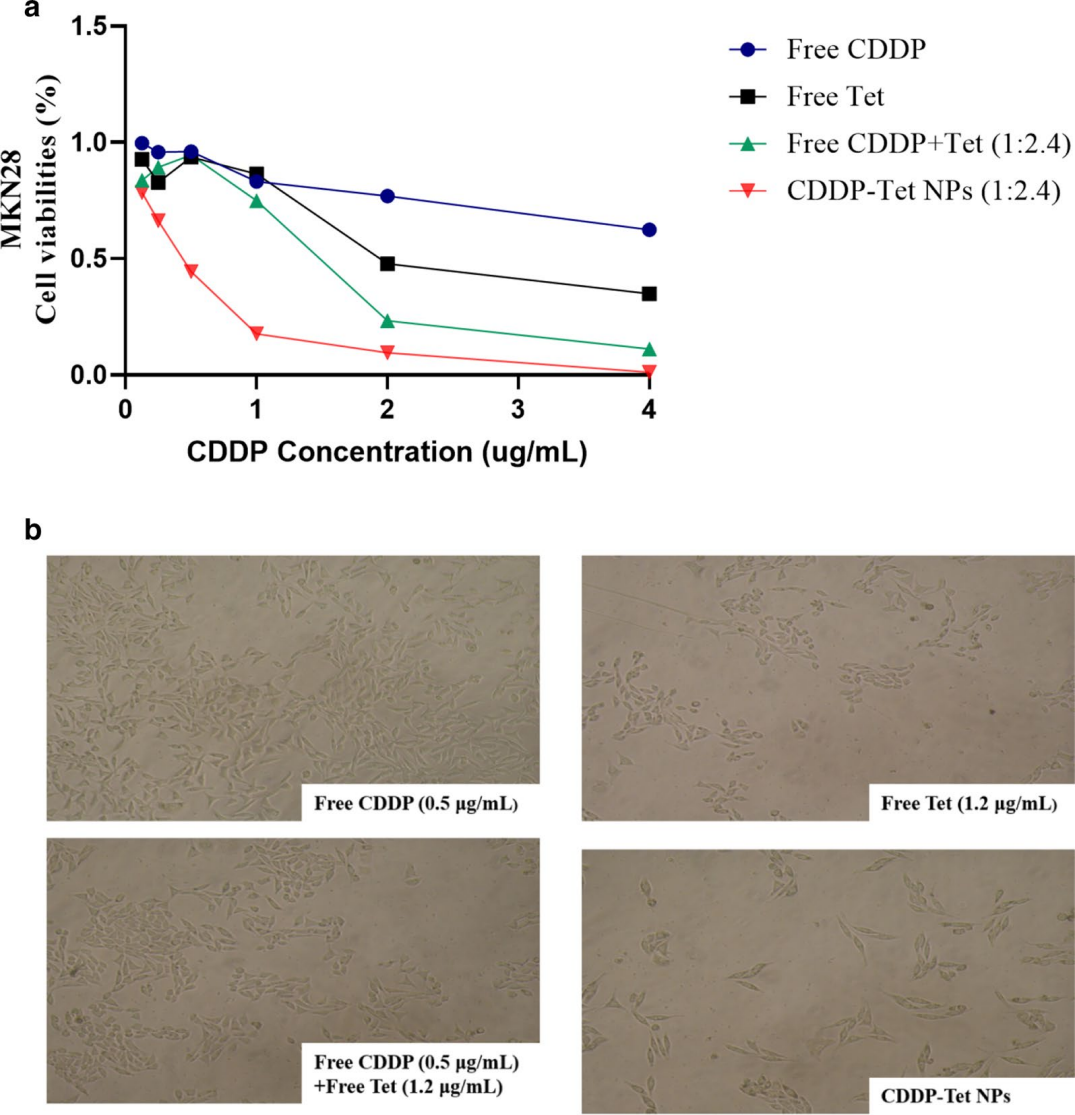
In vitro cytotoxicity of the nanoparticles. **a** Cell viabilities of MKN28 after being co-cultured with drugs for 48 h. The concentration of Tet was 2.4 times as much as the concentration of CDDP. **b** Photographs of MKN28 cells under the light microscopy (200 ×) after being co-cultured with drugs for 48 h

The cytotoxicity of free CDDP, free Tet, free CDDP plus Tet, and CDDP-Tet-loaded NPs were compared in gastric cell line MKN28. The concentration of Tet was 2.4 times as much as the concentration of CDDP. As shown in Fig. 3, the cytotoxicity of CDDP-Tet-loaded NPs was most potent among the four groups. The difference of cytotoxicity between free Tet and the NPs group and three other free-drugs group became prominent with the concentration increased. Similar results were observed in human cervical cancer cells Hela and hepatocellular carcinoma cells H_22_ (Additional file 1: Fig S2a, S2b).

Toxicity study of blank NPs has been conducted in our previous work. In the previous study, the blank of NPs had little toxicity on tumor cell lines, which indicated that blank NPs are of satisfactory biocompatibility [14, 16].

### In Vitro Apoptosis Analysis of CDDP-Tet-Loaded NPs

**Fig. 4:**
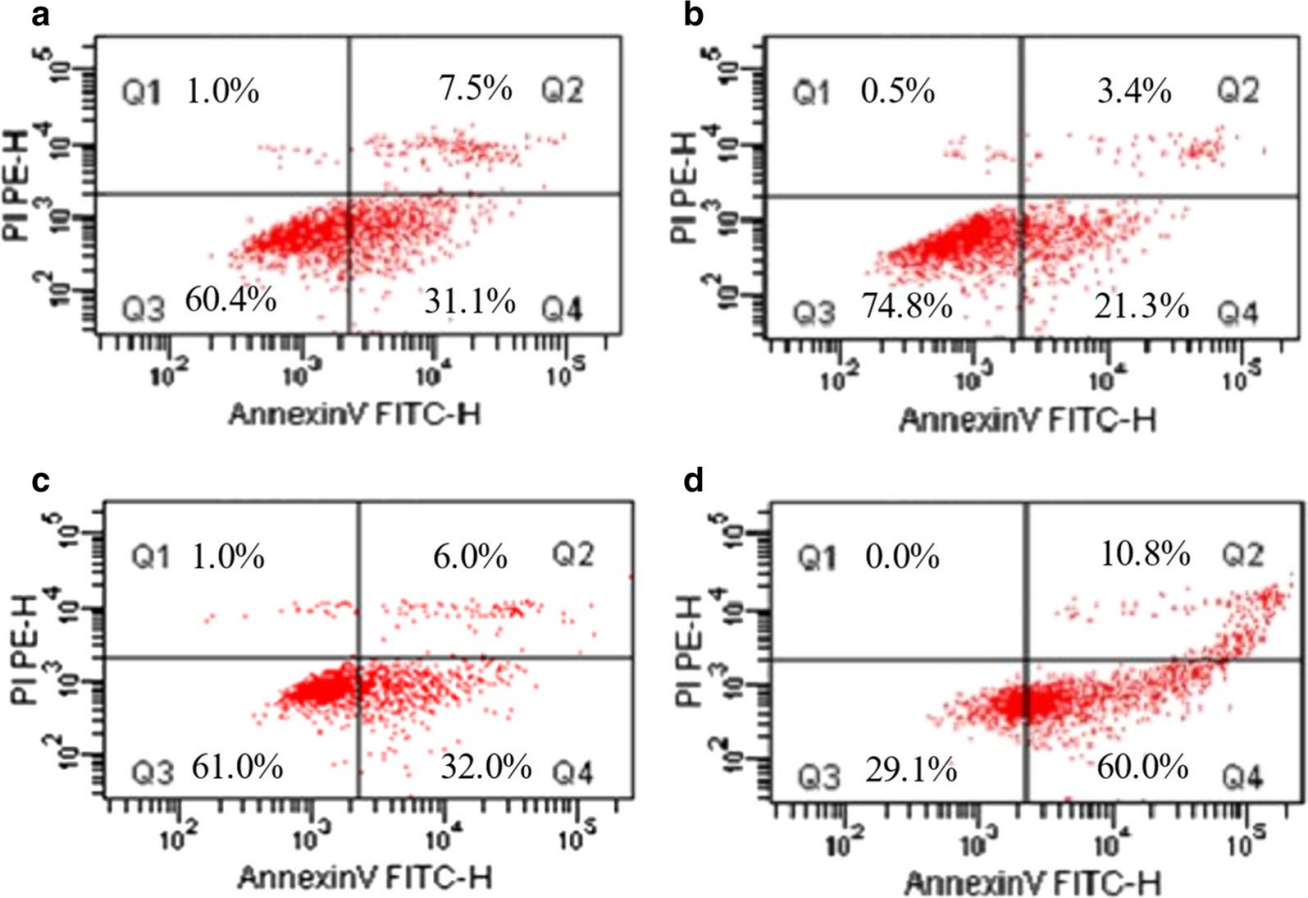
In vitro apoptosis analysis by flow cytometry **a** free CDDP, **b** free Tet, **c** free CDDP + Tet and **d** CDDP-Tet NPs

We measured the impact of 1 μg/mL free CDDP, 2.4 μg/mL free Tet, free CDDP plus Tet (1 μg/mL + 2.4 μg/mL), and CDDP-Tet-loaded NPs(1 μg/mL + 2.4 μg/mL) on apoptotic rate of MKN28 cells after being co-cultured for 48 h. Cell apoptotic rate was calculated as Q2 + Q4 (shown in Fig. 4). The alteration of cells apoptotic rates was similar between free CDDP plus Tet, free CDDP, and free Tet groups (Fig. 4a–c). However, the apoptosis rate of MKN28 cells induced by CDDP-Tet-loaded NPs was significantly higher than three other groups (Fig. 4d).

### Histoculture Drug Response Assay (HDRA)

To evaluate the antitumor effectiveness of the NPs more comprehensively, we evaluated antitumor effects of free CDDP, free CDDP plus Tet, and CDDP-Tet-loaded NPs on H_22_ cell lines using HDRA. As a clinical method to predict chemosensitivity, HDRA simulates practical conditions of tumor tissues more authentically than cytological experiment [19], whose results can be influenced by the microenvironment and microstructure of the tumor tissue such as penetration of drug, the extracellular pH values, interstitial fluid pressure, etc.

**Fig. 5:**
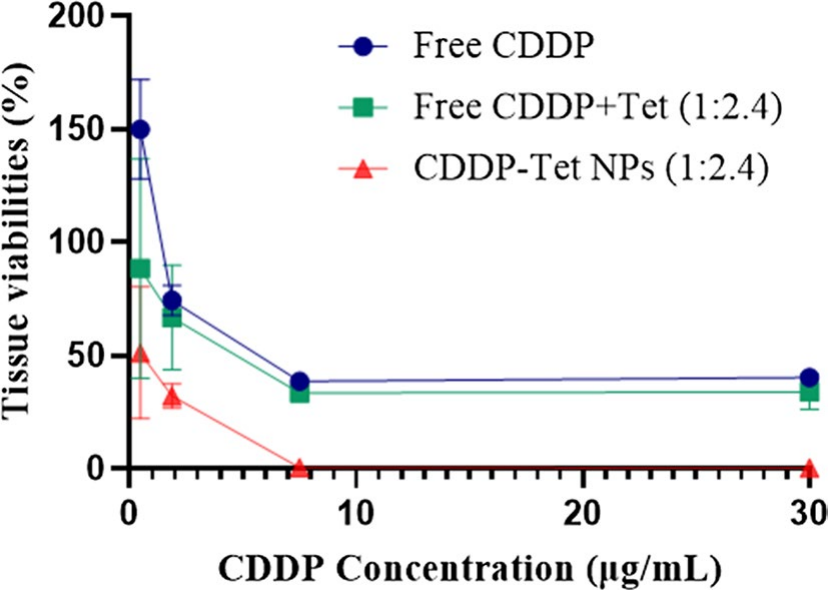
Histoculture drug response assay

The concentration of Tet is 2.4 times as much as the concentration of CDDP. As shown in Fig. 5, at the lowest concentration, the antitumor effect of free CDDP and Tet group was better than that of CDDP yet the effects were similar with the increase of drug concentration. In accord with apoptosis analysis, CDDP-Tet-loaded NPs had a considerably better antitumor effect among three groups at all tested concentrations.

### In Vivo Antitumor Efficacy Analysis

#### Efficacy and Side Effect Evaluation

**Fig. 6:**
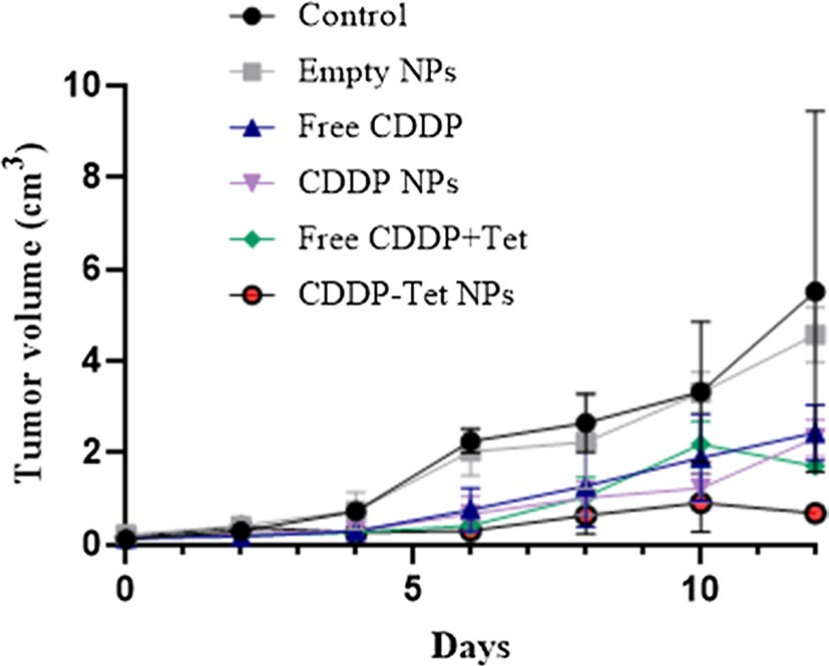
Tumor volume of established H_22_ xenografts in ICR mice during therapy under different treatments. Mice were treated with different strategies on day 0 as shown in the figure: 3 mg/kg of free CDDP, free CDDP together with Tet (CDDP 3 mg/kg + Tet 7.2 mg/kg), and CDDP-Tet-loaded NPs (CDDP 3 mg/kg + Tet 7.2 mg/kg), respectively. Mean ± SD (*n* = 6) of each group was measured

Murine models formed by H_22_ cell line engraftment were treated with 3 mg/kg of free CDDP, free CDDP together with Tet (CDDP 3 mg/kg + Tet 7.2 mg/kg), CDDP-Tet-loaded NPs (CDDP 3 mg/kg + Tet 7.2 mg/kg), respectively. Tumor were obtained 12 days after drug treatment. Tumor sizes were detected every 2 days to determine the most optimum content of drug delivery. As shown in the tumor growth curve (Fig. 6), the tumor growth trend of the control group as well as the blank nanoparticles group were similar but there existed prominent inhibition of tumor in other three groups with drug delivery. Compared with free CDDP group, free CDDP plus Tet group displayed better antitumor efficacy during the first 6 days. However, after 6 days, the difference of tumor inhibition rate between the two groups began to narrow down and after 10 days, free CDDP plus Tet had even worse antitumor effect.

During the first 6 days, the antitumor effects of the free CDDP plus Tet and CDDP Tet-loaded NPs groups were similar. Therefore, murine received CDDP-Tet-loaded NPs showed better antitumor efficacy since day 6 after treatment. Additional file 1: Figure S3a displays different tumor sizes of each group. The tumor volume of CDDP-Tet-loaded NPs group was smallest, which could be regarded as the direct reflection of prominent antitumor effect. In addition to the tumor inhibition capacity, compared with free CDDP or free CDDP plus Tet group, there were less blood vessels located on the surface of the tumor). Similarly, as shown in Additional file 1: Figure S3b, the vascular density was the lowest in the NPs group relative to the control group and free CDDP plus Tet group.

**Fig. 7:**
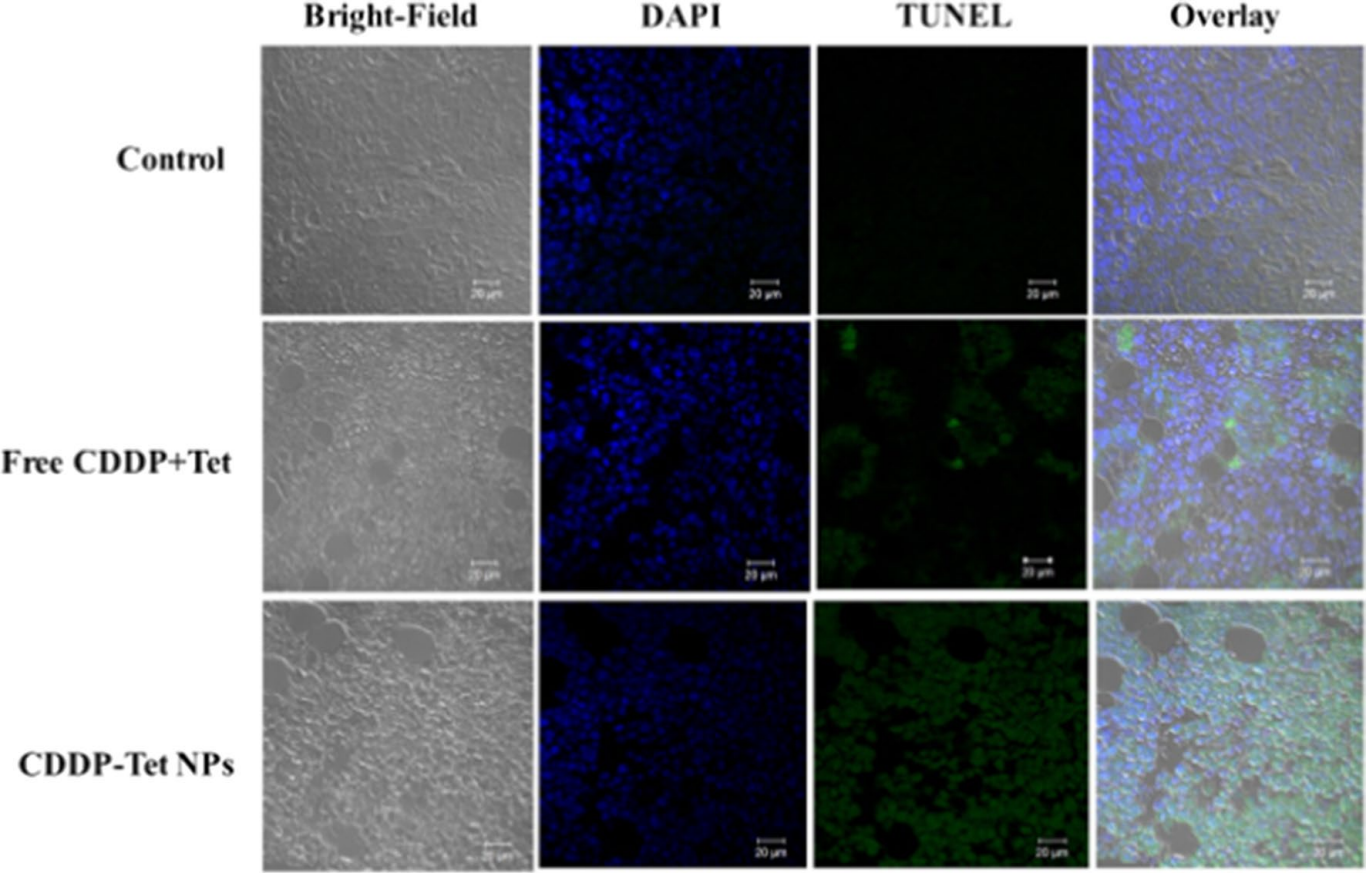
Apoptotic cells were detected by a TUNEL assay (green) and co-stained by nuclear staining DAPI (blue)

To further investigate the ratio of apoptotic cells in tumors tissue in vivo, TUNEL assay was performed for the detection of apoptotic cells. As shown in Fig. 7, the apoptotic cells in tumors can be stained with green fluorescence to indicate apoptosis. The merged images show fewer green fluorescent regions in the control group and the Free CDDP plus Tet group, indicating the presence of fewer apoptotic cells. Moreover, a large number of green fluorescent regions were observed in the group treated with CDDP-Tet NPs, indicating a large amount of apoptotic cells. The results confirmed that CDDP-Tet NPs can promote tumor apoptosis in vivo.

**Fig. 8:**
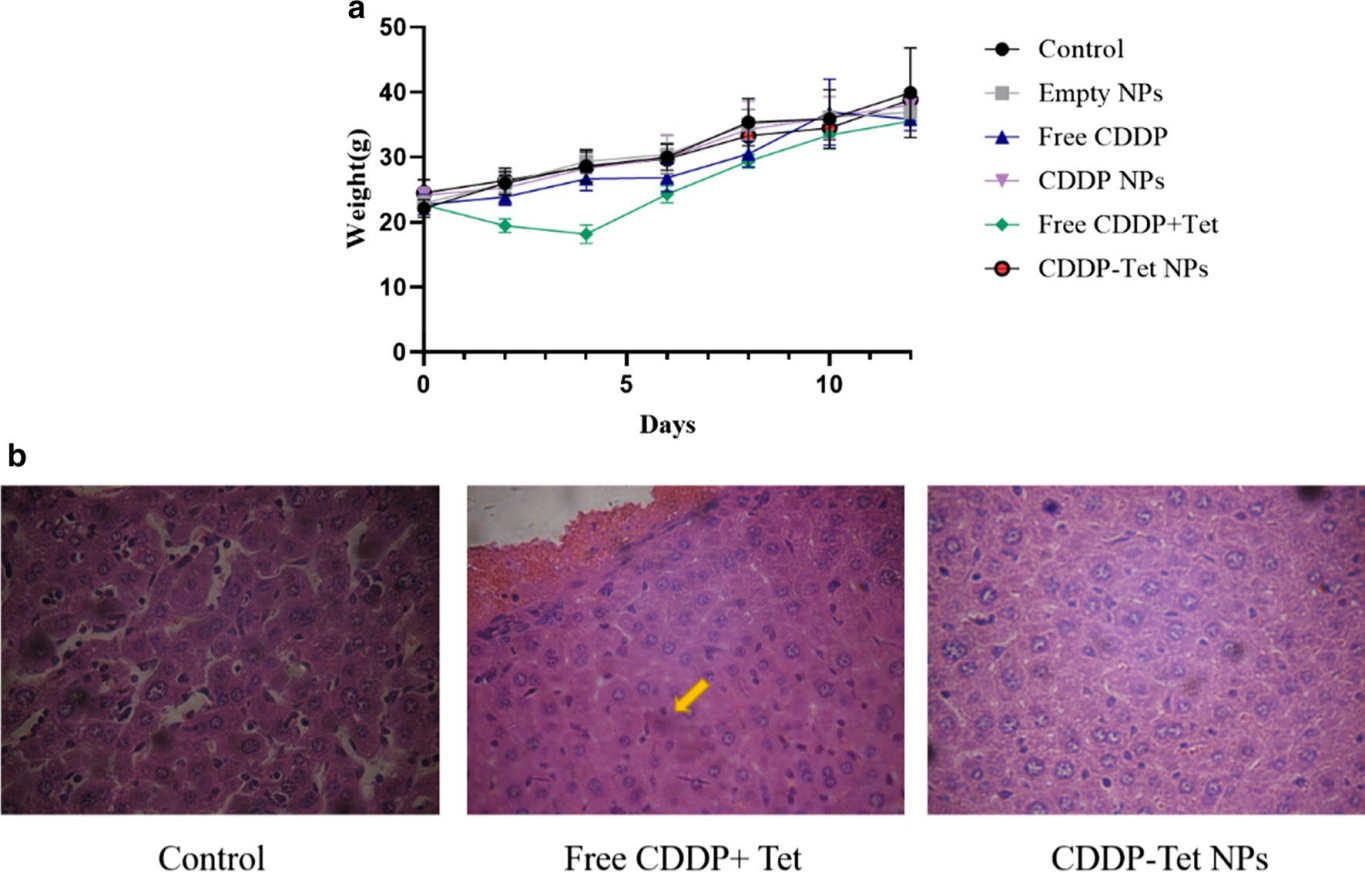
Side effects evaluation **a** body weights of established H_22_ xenografts in ICR mice during therapy under different treatments. **b** Liver specimens stained HE were on light microscopic observation (400 ×)

In addition to better antitumor efficacy than free CDDP and free CDDP plus Tet group, the CDDP-Tet-loaded NPs also displayed fewer side effects. As shown in Fig. 8a, free CDDP and free CDDP plus Tet caused significant weight loss compared with control group, indicating the toxicity with direct drug delivery. The weight change curve of the blank NPs group was similar to that of the control group, suggesting the toxicity of the blank NPs could be disregarded. The weight loss caused by CDDP-Tet-loaded NPs and control groups were comparable during the first 6 days. From day 6 to day 12, murine weight levels in NPs group were slightly lower than that in the control group, but were higher than free CDDP or free CDDP plus Tet group. It is noteworthy that free CDDP plus Tet group contributed to obvious weight loss and decreased the appetite of mice during the first 4 days, which indicates the absorption of drugs was harmful to whole body. By contrast, CDDP-Tet-loaded NPs can be released slowly in tissues and maintain the concentration to a stable degree; therefore, the side effects were obvious lowered compared to direct drug delivery. Then, side effects of the treatment were also evaluated by liver biopsy (Fig. 8b). Compared with the control group, the boundary between the liver cells of the double-drug naked drug group is blurred, some liver cells have ballooned changes, cell bodies shrink, nuclear pyknosis, and increased eosinophilia (yellow arrow), while the double-drug nanoparticle group. The structure of stem cells is normal, the intercellular space is clear, and there is no obvious pathological change. These results suggested there was little impairment caused by NPs delivery.

#### PET–CT

**Fig. 9:**
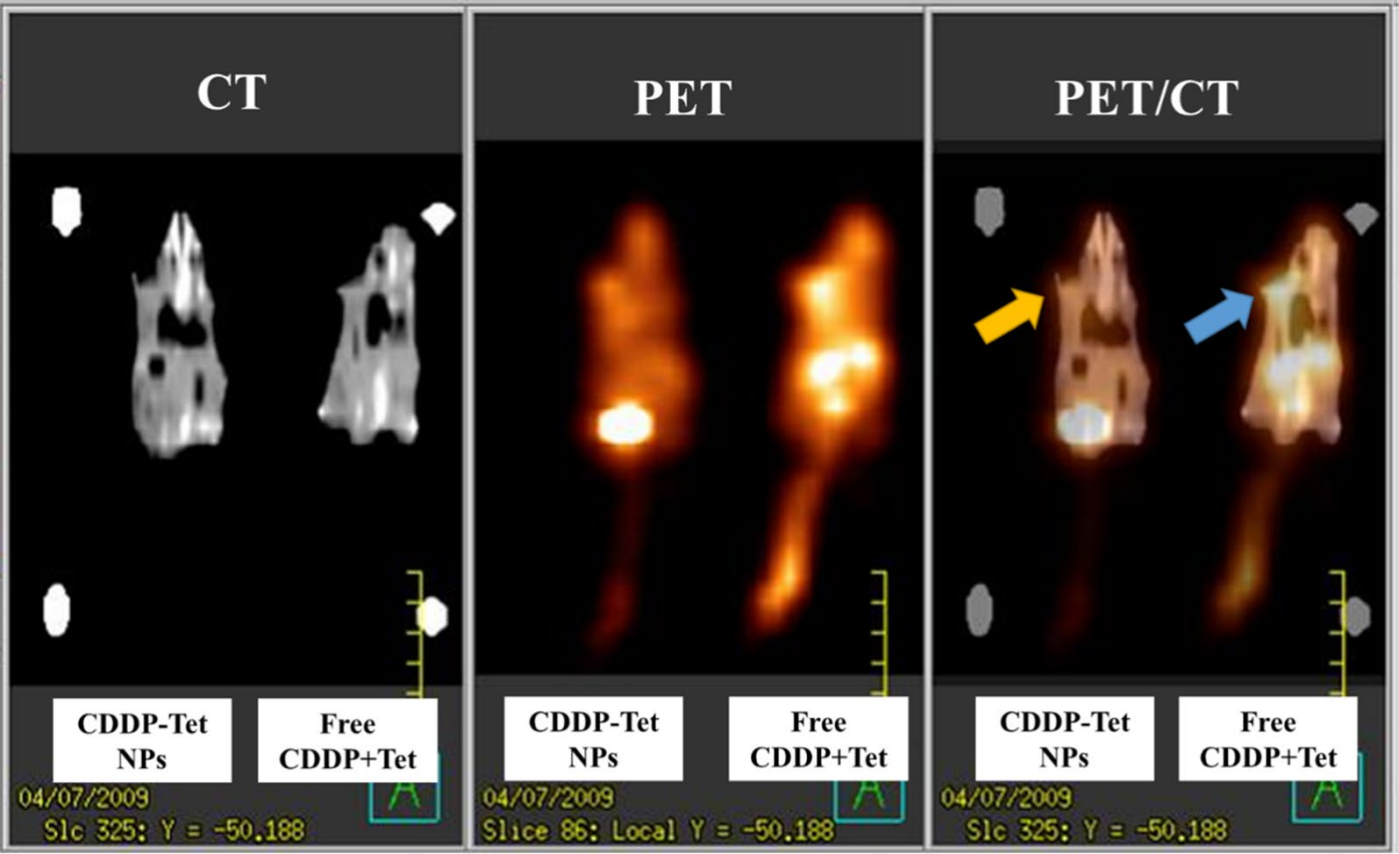
Male ICR mice bearing a subcutaneous H_22_ tumor at the left axillary (arrows). CT, PET and fused PET/CT images are arranged in the figure from left to right. Tumor metabolic rate in free CDDP plus Tet group (blue arrow) was significantly higher than NPs group (yellow arrow)

To better compare in vivo therapeutic effect of each group, mice underwent CT, PET/CT scan at day 6 after treatment. The fusion images of CT and PET scan are displayed in Fig. 9. PET/CT is an efficient method in reflection of metabolic changes through ^18^FDG uptake detection [21]. As shown in CT scan (Fig. 9), the tumor volume of free CDDP plus Tet group and CDDP-Tet-loaded NPs group were comparable (Fig. 9a) but the tumor metabolic rate in free CDDP plus Tet group was significantly higher than NPs group (Fig. 9b). The decreased intensity at the tumor site of the murine received CDDP-Tet-loaded NPs symbolized poor metabolism rate of the tumor, and thereby indicating the capability of NPs to retard tumor growth.

## Discussion

Considering the heterogeneity and complexity of tumor, combination therapy has become a standard strategy in the clinical treatment of tumor [22]. Nevertheless, simple combination of two individual therapeutics can't necessarily reach anticipated effect because of the different physicochemical and pharmacokinetic properties of the two drugs [23]. To deliver different drugs to tumor cells in a synergistic ratio, a combination therapy vehicle has been designed in this study. CDDP is hydrophilic while Tet is hydrophobic, which cause problems for the loading method. In this study, we used the amphiphilic copolymer with PCL as the hydrophobic core and PEG as the hydrophilic corona [14]. By the improved double emulsion method, Tet located in the oil layer and CDDP located in the water layer, the unique structure imparts the NPs with the capacity to simultaneous encapsulation of Tet and CDDP to form NPs. CDDP and Tet are located in different layers of NPs, resulting in low interfering effect and high stability [24].

The combination therapy vehicle loaded with CDDP and Tet can enhance the efficacy of CDDP-Tet combination. Not only in the cellular experiments, CDDP-Tet NPs also significantly inhibit tumor tissue viabilities in the HDRA assays, validating the anti-tumor effect of the NPs in the model which take into account of tumor microenvironment [19]. As to in vivo study, antitumor efficacy was observed in tumor volume change, which demonstrated that CDDP-Tet NPs effectively suppressed tumor growth with lower proliferation level. ^18^FDG-PET/CT imaging revealed that the glucose metabolism of the tumors in the CDDP-Tet group was inhibited more prominently and early by inducing higher apoptosis level of tumors, which was confirmed in the immunofluorescence assays [21]. Compared with free CDDP plus Tet, CDDP-Tet NPs are faster and safer to take effect, which can be explained by the three mechanisms as follows.

Firstly, as a lipid-soluble drug, Tet can hardly distribute in the ECM and therefore rarely diffuse around tumor cells [9]. Located in the oil phase of NPs, Tet is endowed with better solubility and bioavailability, reaching the tumor sites in the same synergistic ratio as CDDP. Furthermore, CDDP, which used to have systemic side effect, once carried by the nanoparticle, can easily reach the interstitium of tumor tissues from leaky tumor blood vessels and be held within the tumor on account of pressure made by destitute lymphatic drainage [25]. The more CDDP tumor tissues hold, the less damage will be done to normal organs. As a result of passive targeting strategies, CDDP-Tet NPs are much safer than free CDDP plus Tet, which can be observed in the murine body weight changes and liver biopsies.

Drug resistance is regarded as one of the greatest challenges in cancer treatment, not only because of genetic changes at the level of a single cell, but also due to tumor tissues and microenvironment [26]. On one hand, Tet is alkaline, so it will be protonated in the acidic tumor microenvironment and thus can’t cross the electronegative tumor cytomembranes, which is called pH-induced physiological drug resistance [27]. On the other hand, large distances between blood vessels in solid tumors and high interstitial fluid pressure contribute to limited distribution of CDDP and Tet [28]. Carried by the nanovehicle, Tet can enter tumor cells via endocytosis without influence of tumor microenvironment, overcoming the pH-induced physiological drug resistance. The small size of nanocarriers allows them to enter tumor vasculature and preferentially accumulating at the tumor site in vivo [29, 30].

In summary, this study represents an example of delivering a chemotherapeutic drug along with a chemosensitizer simultaneously. Carried by the NPs, both drugs are targeted to tumor site passively, reducing systemic toxicity. Besides, NPs offer a solution to physiological drug resistance by helping the chemosensitizer enter tumor cells more and faster, which play an important role in improving the efficacy of tumor chemotherapy. Therefore, we believed that this PEG–PCL block copolymeric NPs could be a promising carrier for combined chemotherapy.

## Conclusions

Based on our previous studies [8, 14, 16], this paper investigated the application of PEG–PCL/HO-PCL NPs for delivery of the combination of two drugs with different physicochemical properties. For in vitro studies, NPs exhibited superior antitumor effect with great biocompatibility. Enhanced antitumor efficacy was observed in tumor volume change and ^18^FDG-PET/CT Imaging as to in vivo study in murine model. The mice in NPs group also exhibited reduced side effects. Additionally, the apoptosis rate of tumor cells was promoted by NPs in both in vitro and in vivo studies. In summary, this block copolymeric NPs could be a promising carrier for the delivery of Cisplatin–Tetradrine combinations and other combinations, with enhanced antitumor effect and reduced toxicity, for the treatment of cancer.

## Supplementary Information

**Additional file 1.** **Fig. S1.** a. Transmission electron microscope images of CDDP-Tet NPs. b. Atomic force microscopy images of CDDP-Tet NPs. **Fig. S2.** In vitro cytotoxicity of the nanoparticles. a. Cell viabilities of H22 after being co-cultured with drugs for 48 hours. The concentration of Tet was 2.4 times as much as the concentration of CDDP. b. Cell viabilities of Hela after being co-cultured with drugs for 48 hours. The concentration of Tet was 2.4 times as much as the concentration of CDDP. **Fig. S3.** a The specimens of tumors on murine models at the endpoint of the experiment. b Tumor specimens stained HE were on light microscopic observation (200X). **Table S1.** Characteristics of the optimal CDDP-Tet loaded PEG-PCL copolymeric NPs. **Table S2.** Drug loading content and loading efficiency of CDDP-Tet loaded PEG-PCL copolymeric NPs.

## Data Availability

The datasets used and/or analysed during the current study are available from the corresponding author on reasonable request.

## Abbreviations

Tet: Tetradrine
CDDP: Cisplatin
NP: Nanoparticle
PEG–PCL: Poly(ethyleneglycol)–polycaprolactone
HO-PCL: Polycarprolactone
ECM: Extracellular matrix
PEG: Polyethylene glycol
RES: Reticuloendothelial system
DMF: Dimethylformamide
SPF: Specific pathogen-free
Mn: Number-average molecular weight
Mw: Weight-average molecular weight
DLS: Dynamic light scattering
