# Supplementary material for: Biocompatible Nanoparticles as a Platform for Enhancing Antitumor Efficacy of Cisplatin–Tetradrine Combination
Source: Nanoscale Res Lett. 2021 Apr 14;16:61. doi: 10.1186/s11671-021-03511-4 (PMC8046896; doi:10.1186/s11671-021-03511-4)
Supplement: Supplementary file 1 — Additional file 1. Fig. S1. a. Transmission electron microscope images of CDDP-Tet NPs. b. Atomic force microscopy images of CDDP-Tet NPs. Fig. S2. In vitro cytotoxicity of the nanoparticles. a. Cell viabilities of H22 after being co-cultured with drugs for 48 hours. The concentration of Tet was 2.4 times as much as the concentration of CDDP. b. Cell viabilities of Hela after being co-cultured with drugs for 48 hours. The concentration of Tet was 2.4 times as much as the concentration of CDDP. Fig. S3. a The specimens of tumors on murine models at the endpoint of the experiment. b Tumor specimens stained HE were on light microscopic observation (200X). Table S1. Characteristics of the optimal CDDP-Tet loaded PEG-PCL copolymeric NPs. Table S2. Drug loading content and loading efficiency of CDDP-Tet loaded PEG-PCL copolymeric NPs. [file 11671_2021_3511_MOESM1_ESM.docx]

Supplementary material

**Biocompatible Nanoparticles as a Platform for Enhancing Antitumor Efficacy of Cisplatin–Tetradrine Combination**

FangcenLiu^1,2,†^, XinyueWang^2 ,†^, QinLiu^2^, HuanZhang^3^, LiXie^2^, QinWang^2^, LinLi^1^, RutianLi^2 ,^*

*1Department of Pathology, Nanjing Drum Tower HospitalThe Affiliated Hospital of Nanjing University Medical SchoolNanjingChina*

*2The Comprehensive Cancer Centre of Nanjing Drum Tower HospitalThe Affiliated Hospital of Nanjing University Medical SchoolNanjingChina*

*3Center for Personalized MedicineLinköping University58183LinköpingSweden*

*Received: 25 December 2020 / Accepted: 21 March 2021*

______________________

^*^Corresponding author.

E-mail: [rutianli@nju.edu.cn](mailto:Emailrutianli@nju.edu.cn)

^†^These two authors contributed equally to this work.

**Characterization of NPs**

**Size of NPs**

Mean diameter and size distribution were measured by photon correlation spectroscopy (DLS) with a Brookheaven BI-9000AT instrument (Brookheaven Instruments Corporation, USA). Zeta potential was measured by the laser Doppler anemometry (Zeta Plus, Zeta Potential Analyzer, Brookhaven Instruments Corporation, USA). All measurements were performed at 25℃. The reported values were determined from at least three measurements.

**Morphology Studies**

Morphological examination of the nanoparticles was conducted using JEM-100S (Japan) transmission electron microscope (TEM). One drop of nanoparticle suspension was placed on a copper grid covered with nitrocellulose membrane and air-dried before observation.


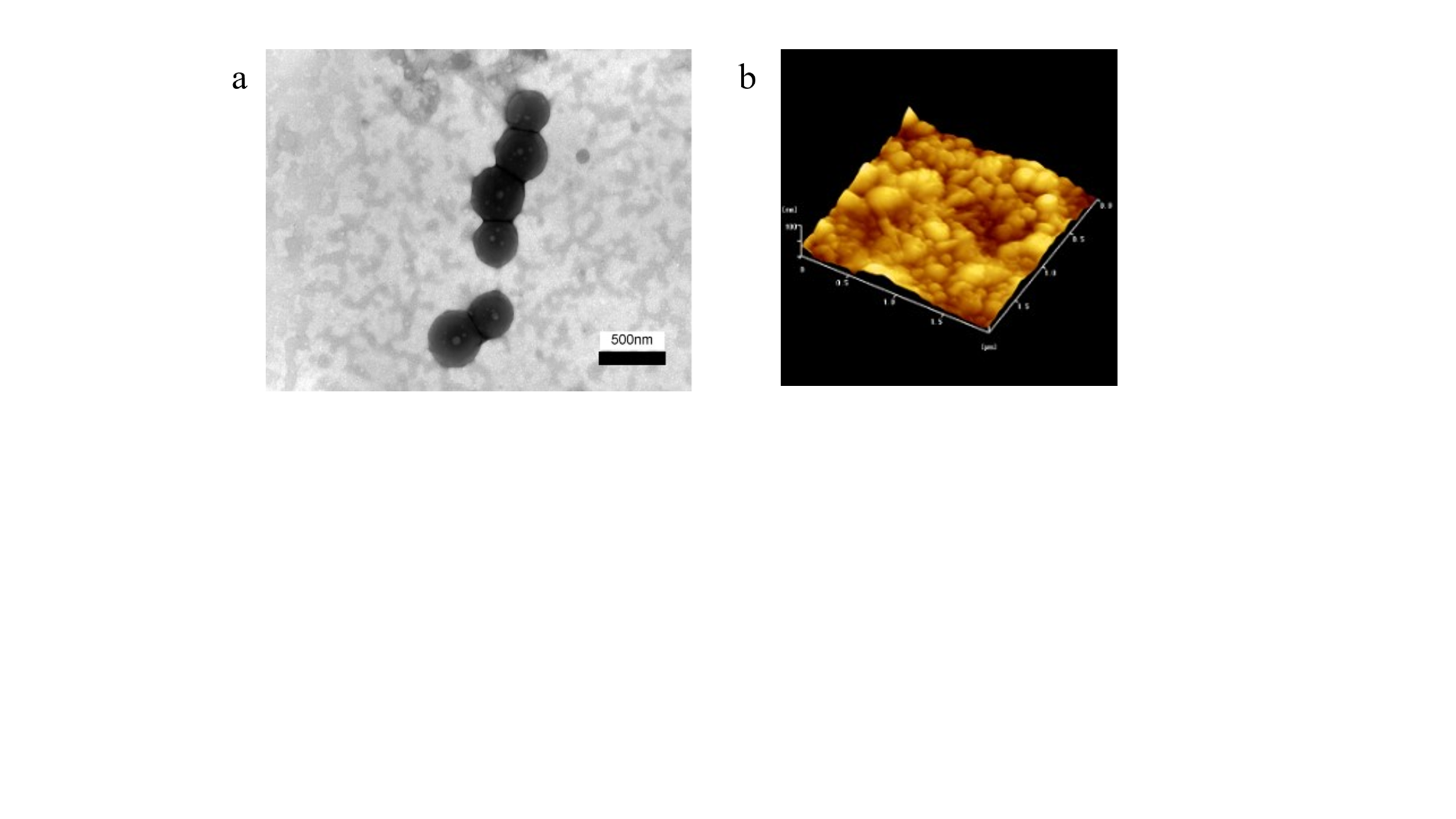


FigS1. **a**.Transmission electron microscope images of CDDP-Tet NPs **b**. Atomic force microscopy images of CDDP-Tet NPs


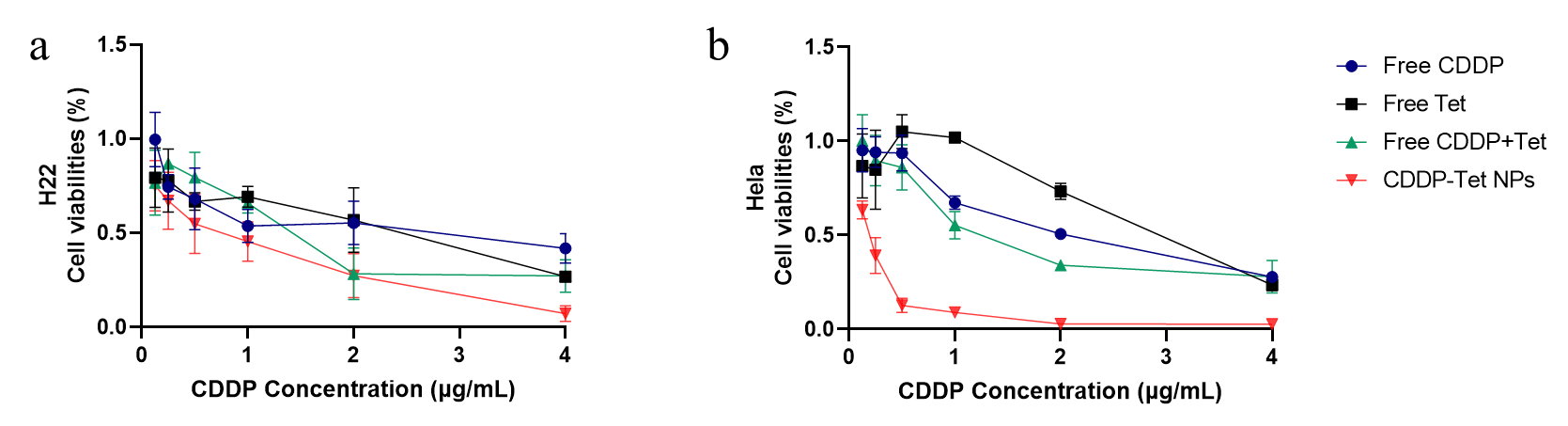


Fig S2. In vitro cytotoxicity of the nanoparticles. **a**. Cell viabilities of H22 after being co-cultured with drugs for 48 hours. The concentration of Tet was 2.4 times as much as the concentration of CDDP. **b**. Cell viabilities of Hela after being co-cultured with drugs for 48 hours. The concentration of Tet was 2.4 times as much as the concentration of CDDP.


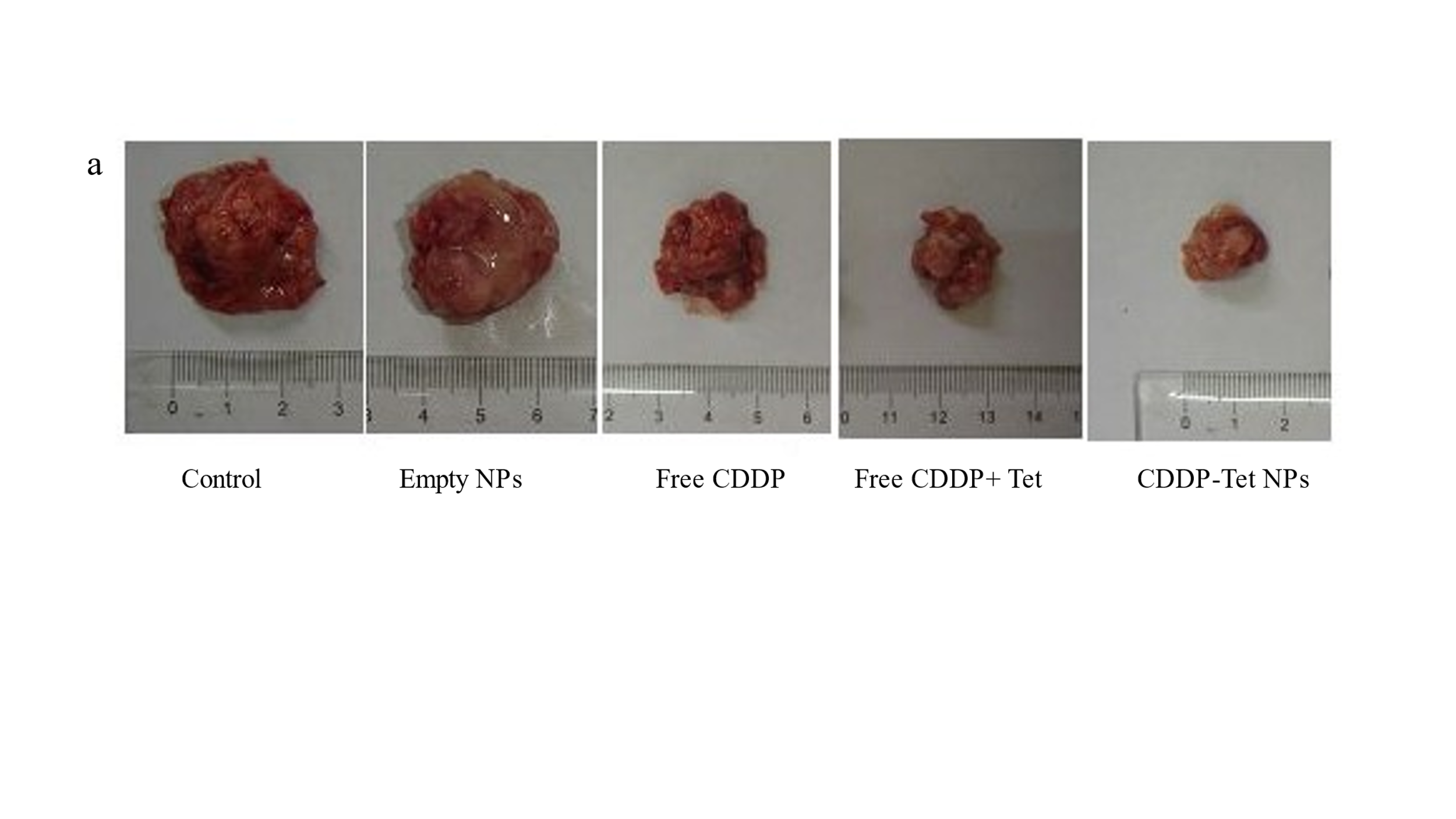


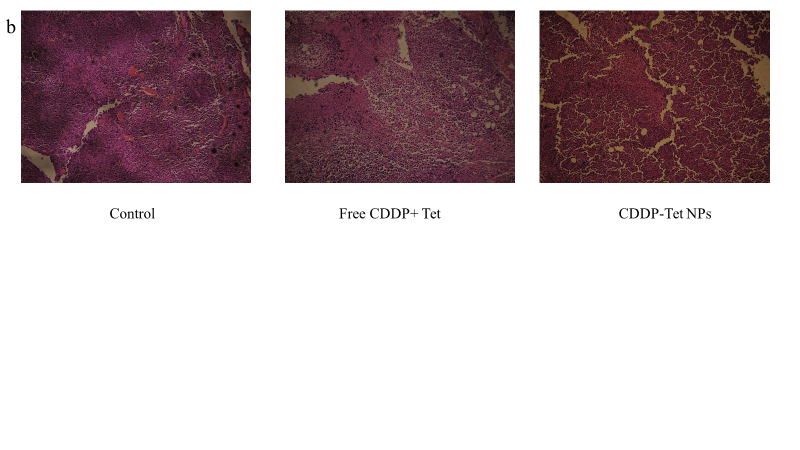


Fig S3. **a** The specimens of tumors on murine models at the endpoint of the experiment. **b** Tumor specimens stained HE were on light microscopic observation (200X).

Table S1. Characteristics of the optimal CDDP-Tet loaded PEG-PCL copolymeric NPs

| NPs | Mn | Hydrophobic ratio (%) | Diameter (nm) | Polydispersity |
| --- | --- | --- | --- | --- |
| mPEG-PCL | 25826 | 83.11 | 285.8±4.2 | 0.054±0.085 |
| HO-PCL | 21038 | / | / | / |
| CDDP-Tet loaded NPs | / | / | 359.1±5.3nm | 0.231±0.00 |

NPs = Nanoparticles; Mn = Number-average molecular weight measured by gel permeation chromatography

Table S2. Drug loading content and loading efficiency of CDDP-Tet loaded PEG-PCL copolymeric NPs

| Drug | Drug loading content | Loading efficiency |
| --- | --- | --- |
| CDDP | 3.32% | 65.3% |
| Tet | 7.45% | 27.9% |
